# Birmingham and Midland Hospital for Women

**Published:** 1905-09-30

**Authors:** 


### BIRMINGHAM AND MIDLAND HOSPITAL FOR WOMEN.

FINE weather attended the opening of the new buildings of the Birmingham and Midland Hospital for Women at Sparkhill last week. The ceremony was performed by Mrs. J. S. Nettlefold in the presence of a large gathering of friends.

The site of the new hospital is about one and three-quarter acres in extent, with frontages in Showell Green Lane and Park Road, and it is immediately adjacent to the Sparkhill Recreation Ground. Accommodation is provided for 48 patients, as compared with 24 in the old building. The wards are contained in two wings, which are two stories in height, and have as their main feature four large wards, each containing ten beds. There are also four small wards each containing two beds. The wards in the upper story have open balconies on three sides, and are supplied with an iron emergency staircase. Each block rests upon a basement of open arches, and everywhere there is ample cross-ventilation.

Several gifts have been received, a notable one being that of Messrs. Charles Cartwright and Sons, who presented the beds.

The cost of the new hospital, including the site, is £45,000, and it is very satisfactory to know that the whole of this amount has been provided already so that the institution can make a clear start on its new career of usefulness unencumbered by building debts.

The architects are Messrs. Martin and Martin.

